# Salivary and pellicle proteome: A datamining analysis

**DOI:** 10.1038/srep38882

**Published:** 2016-12-14

**Authors:** Hardy Schweigel, Michael Wicht, Falk Schwendicke

**Affiliations:** 1 Department of Clinical Research, DMG Dental-Material Gesellschaft, Hamburg, Germany; 2 Polyclinic of Operative Dentistry and Periodontology, University of Cologne, Kerpener Straße 32, 50931 Cologne, Germany; 3 Operative and Preventive Dentistry, Charité – Universitätsmedizin Berlin, Berlin, Germany

## Abstract

We aimed to comprehensively compare two compartmented oral proteomes, the salivary and the dental pellicle proteome. Systematic review and datamining was used to obtain the physico-chemical, structural, functional and interactional properties of 1,515 salivary and 60 identified pellicle proteins. Salivary and pellicle proteins did not differ significantly in their aliphatic index, hydrophaty, instability index, or isoelectric point. Pellicle proteins were significantly more charged at low and high pH and were significantly smaller (10–20 kDa) than salivary proteins. Protein structure and solvent accessible molecular surface did not differ significantly. Proteins of the pellicle were more phosphorylated and glycosylated than salivary proteins. Ion binding and enzymatic activities also differed significantly. Protein-protein-ligand interaction networks relied on few key proteins. The identified differences between salivary and pellicle proteins could guide proteome compartmentalization and result in specialized functionality. Key proteins could be potential targets for diagnostic or therapeutic application.

The oral cavity comprises hundreds of proteins with a large range of biological functions like immune defense and biofilm homeostasis, nutrient decomposition and remineralization of dental hard tissues^1^,^2^. These proteins are characterized by molecular features, which are abstractly expressed by scores like aliphatic index, hydrophaty, instability index, net charge and isoelectric point. Similarly, proteins have unique amino acid sequences which affect their structure, function, binding capability, functionalization via glycosylation and phosphorylation as well as interaction with other proteins, ligands or bacteria^3^. Thus, to understand the organization and function of a proteome and to identify possible targets for medical diagnostics or therapy, the individual properties and the resulting relevance of a protein in a specific environment needs to be explored^4^,^5^. Such deeper understanding of all proteins in a proteome would allow to identify possible physico-chemical, structural or functional patterns, which might facilitate a deeper comprehension of the local protein biology and aid translation of obtained findings to other proteomes^4^,^6^.

In the oral cavity, there is in fact not one, but a number of proteomes. With regards to dental hard tissues, two proteomes are relevant; the salivary proteome and the acquired enamel pellicle proteome. Both have been described in a number of studies^7^,^8^,^9^. While the salivary proteome is of high complexity and regulates soluble signaling molecules or ions as well as the oral defense via antibodies, the pellicle proteome is smaller and acts as substrate protector, lubricant and regulator of dental hard tissue mineral homeostasis, while also presenting binding motifs for bacterial surface receptors and thereby enabling the colonization of teeth by bacteria^10^,^11^,^12^.

Unsurprisingly, the pellicle proteome seems to constitute a subpopulation of the saliva proteome. The local and probably functional compartmentalization of both proteomes is currently not fully understood^13^. Therefore, the present study aimed to gain insight into the protein properties leading to this compartmentalization, thereby allowing to identify functional differences and patterns on protein and proteome level with possible relevance for clinical or translational application. Given that single studies are usually unable to allow full statistical exploration of a larger number of properties and additionally suffer from limited reliability, a systematic review and datamining approach was taken.

## Results

### Review findings

**Figure 1:**
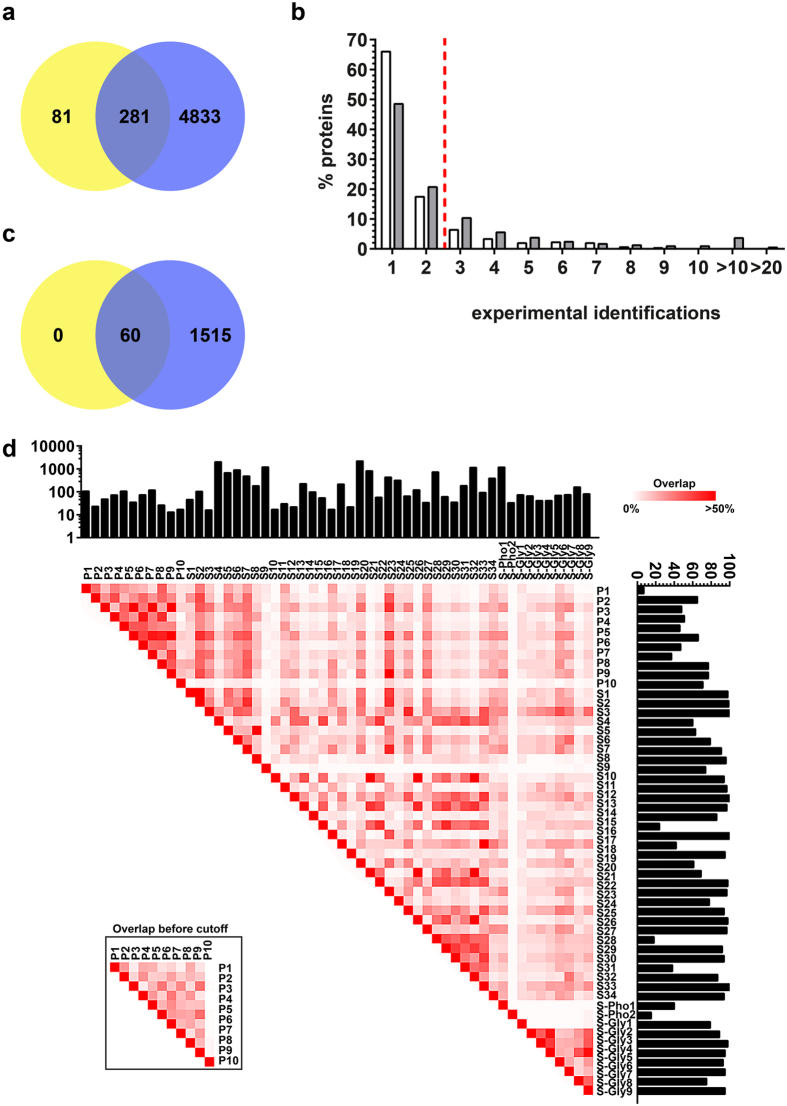
The preliminary and the final dataset. **(a)** The preliminary dataset included all extracted proteins, with poor overlap between salivary (blue) and pellicle proteins (yellow) (this overlap should be much higher given that pellicle proteins stem from the saliva). **(b)** Proportion of proteins identified n-times in the pellicle (white) or saliva (grey). The majority of proteins in both proteomes was reported only once or twice. The dotted red line indicates the applied stringency cutoff for inclusion in the final database. **(c)** After applying the cutoff of three independent experimental identifications, the final database included 1,575 proteins. In this set, all pellicle proteins (yellow) were also reported for the saliva (blue). **(d)** A heatmap displays the relative agreement of proteins reported in different studies (i.e. the % of proteins identified in one compared with the other study after applying the cutoff). The number of originally reported proteins (before cutoff) is shown in the upper bar chart and the proportion of proteins included in the final database (per original number of reported proteins) is shown in the bar plot on the right. To demonstrate the effect of cutoff application, relative agreement between reported pellicle proteins before applying the cutoff is shown in the inbox. P: Pellicle, S: Saliva, S-Pho: phosphorylated proteins of saliva, S-Gly: Glycosylated proteins of saliva.

Our systematic review identified 43 articles reporting proteomic data on salivary proteins and 11 articles on the acquired enamel pellicle. Included studies indexed a mean of 630 (26/6,830) (mean [min/max]) proteins for saliva and 85 (17/223) proteins for the pellicle. The resulting preliminary dataset included a total of 5,228 proteins (4,833 uniquely found in saliva, 81 uniquely found in the pellicle, 281 found in both) (Fig. 1a). The majority of the proteins were reported only once or twice (Fig. 1b). Using three independent experimental identifications as stringency cutoff for inclusion, a total of 1,515 proteins remained in the salivary proteome and 60 in the pellicle proteome (30.2% of the originally identified proteins; 30.8% in saliva and 16.6% in pellicle proteome) (Fig. 1c, Supplementary Table 1). All proteins in the pellicle proteome were also reported in the salivary proteome. The mean overlap of proteins reported by different studies was 10.8% (0.0/84.2%) (mean [min/max]) for saliva and 24.9% (0.0/62.3%) for the pellicle (Fig. 1d).

The resulting database was validated against proteome data reported for the salivary glands (the main source of oral proteins), as recorded by two independent global data resources using immunohistological^14^ and mass spectrometric identification^15^. We confirmed 87.1% of the included proteins to have been reported there at least once, but only 49.8% of the excluded proteins (Supplementary Fig. 1). Additionally, we did not find indication for possible selection bias via molecular weight or experimental signal intensity (Supplementary Fig. 2).

### Physico-chemical proteome characteristics

**Figure 2:**
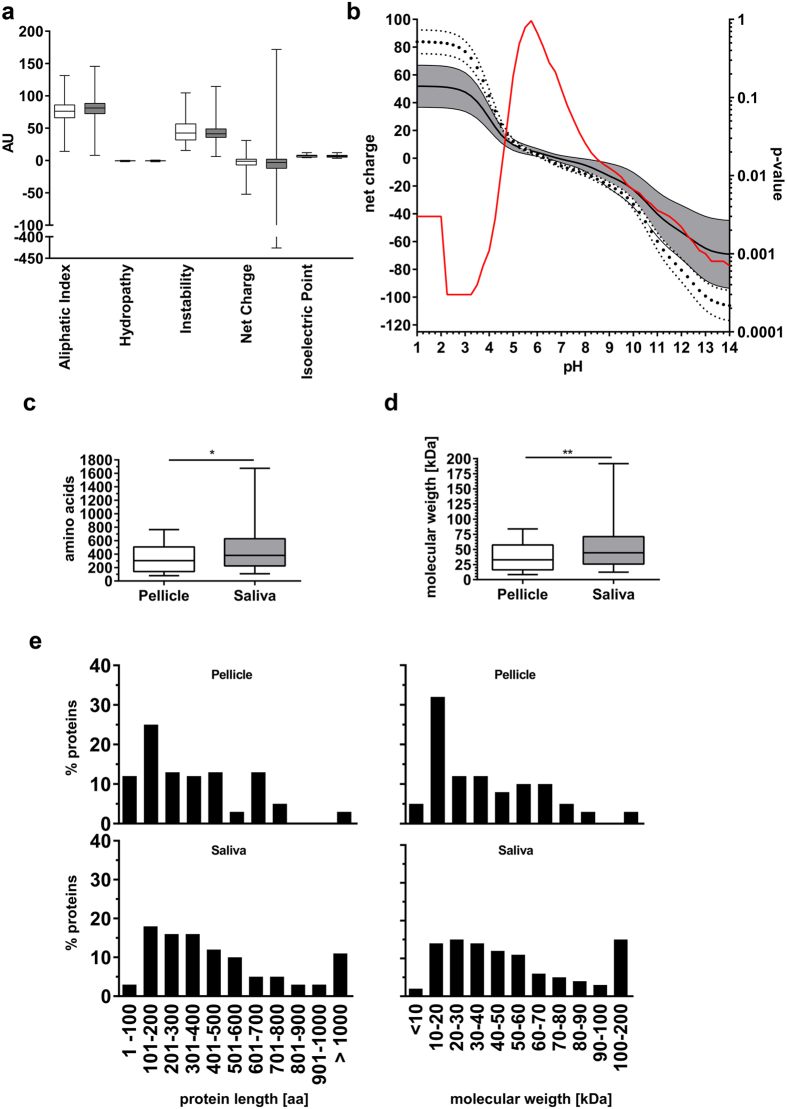
Physico-chemical properties of pellicle and salivary proteins. **(a)** The box plots represent the median (box: 25^th^/75^th^ percentiles, whiskers: minimum/maximum) physico-chemical properties of the pellicle (white) and salivary (grey) proteins. No statistically significant difference between both proteomes was found. **(b)** Mean net charge (±95% CI) of both proteomes at different pH. Pellicle proteins (white, dotted confidence strand) showed a higher positive net charge under acidic conditions (<pH 4.25) and higher negative charge under basic conditions (>pH 10.5). The red line indicates the calculated p-values for each pH. **(c–e)** Analysis of molecular weight and length of protein chains of pellicle (white) and salivary (grey) proteins. Pellicle proteins were significantly smaller than salivary proteins. The major fraction of pellicle proteins was smaller than 30 kDa or shorter than 300 amino acids. Large proteins (>100 kDa/>900 amino acids) were more often found in saliva. Statistical comparison of proteomes was performed using Mann-Whitney-U test: *p < 0.01, **p < 0.001.

To gain deeper insight into the specific features of both proteomes, we first assessed the physico-chemical protein properties like aliphatic index, hydrophaty, instability index, net charge and isoelectric point. We found no significant differences (p > 0.2605; Mann-Whitney-U-test) between proteins of the saliva and the pellicle for the investigated properties (Fig. 2a).

As the oral environment is exposed to physiologically varying pH-values, we calculated the net charge for every protein per proteome at incremental pH-steps between pH 1 and 14. We found significant differences between the two proteomes (Fig. 2b), with pellicle proteins having a higher mean net charge under extreme pH conditions than salivary proteins (pH 1.00–4.25: p = 0.0015; pH 9.50–14.00: p = 0.0033).

Next, we investigated the protein size in both proteomes. In general, pellicle proteins were significantly smaller and lighter (p = 0.0009 for molecular weight, p = 0.0042 for molecular length) than salivary proteins. Whereas half of the proteins of the pellicle were smaller than 30 kDa, larger proteins (>100 kDa) were almost exclusively found in the salivary proteome (Fig. 2c–e).

### Analysis of amino acid distribution and protein structures

**Figure 3:**
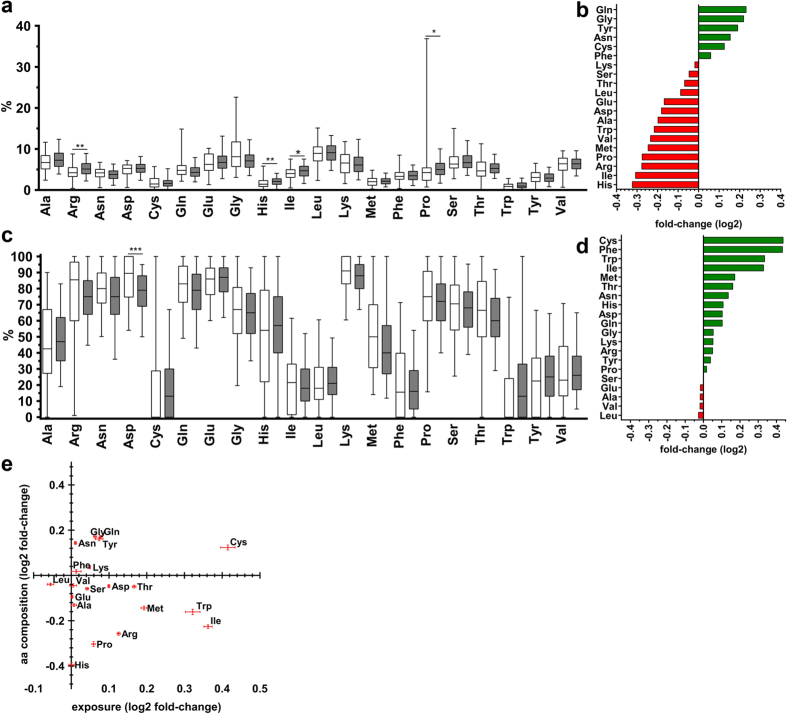
Observed amino acid frequency and predicted solvent molecular surface exposure of amino acids. **(a)** Four amino acids (Arg, His, Ile, Pro) were significantly more or less expressed in the pellicle (white) than in the saliva (grey). **(b)** Mean fold-changes (log2) of amino acid expression in the pellicle compared with saliva. Proteins of the pellicle included Gln, Gly, Tyr, Asn, Cys and Phe more often than salivary proteins, whereas expression frequency of all other amino acids was lower in pellicle than salivary proteins. **(c)** Summary of predicted amino acid exposure in proteins of the pellicle (white) and the saliva (grey). A significant difference was found for Asp only. **(d)** Predicted exposure of amino acids was higher in the pellicle than salivary proteins for all but five amino acids (Ser, Glu, Ala, Val, Leu). **(e)** Combined results (mean ± 95% CI [log2]) did not reveal a single amino acid which was both differently expressed and differently exposed. Mann-Whitney-U test was applied for statistical comparison: *p < 0.01, **p < 0.001, ***p < 0.0001; aa  =  amino acid.

The amino acid distribution differed significantly between both proteomes, with Histidine, Isoleucine, Proline and Arginine being underrepresented in the pellicle (p < 0.01, Fig. 3a,b).

The solvent molecular surface exposure of the specific amino acid residues differed significantly for Asparagine (p < 0.0001) (Fig. 3c,d). Sixteen amino acids were proportionally over-exposed in the pellicle compared with the saliva (with Cysteine, Phenylalanine and Tryptophan as the most over-exposed), while these differences did not reach statistical significance (p > 0.02). Four amino acids (Leucine, Valine, Alanine and Glutamine) were under-exposed, again without statistical significance. When assessing the combination of amino acid distribution and exposure, there was no single amino acid which differed greatly in not one but both properties in the pellicle versus the saliva (Fig. 3e).

**Figure 4:**
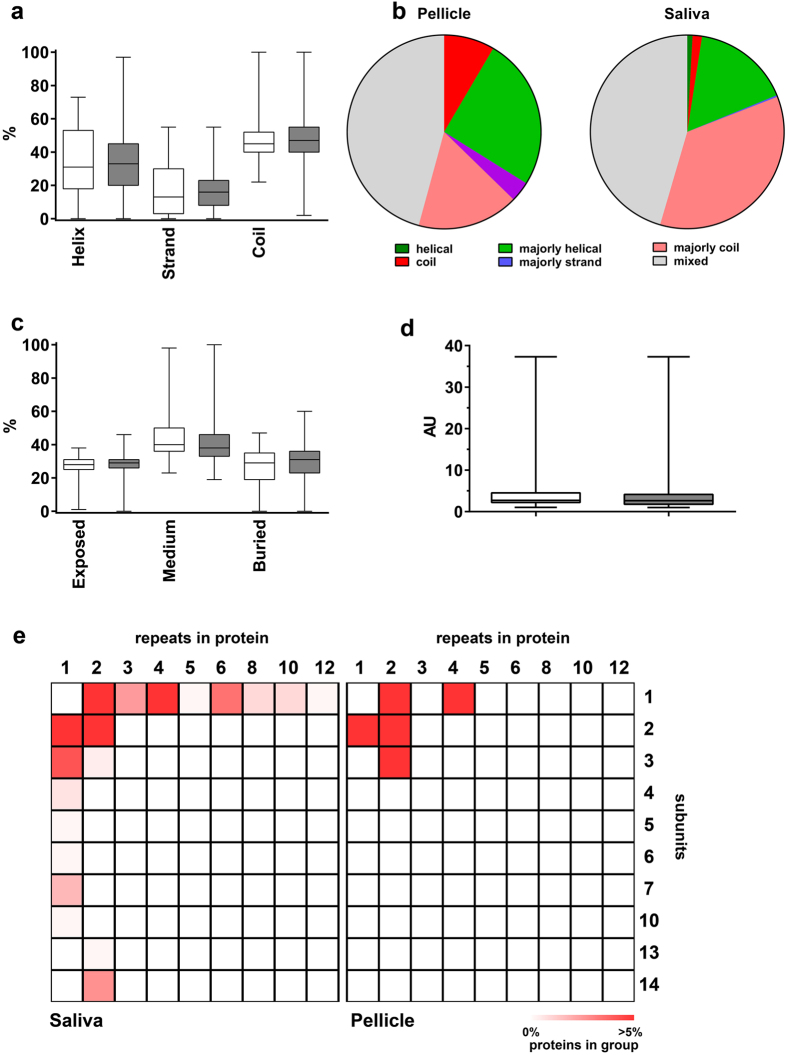
Predicted secondary structure motives, solvent surface access, molecular shape and organization of pellicle and salivary proteins. (**a**) Typical structures like alpha helices, beta sheets and coils were not predicted significantly different for both proteomes (pellicle: white; saliva: grey). (**b**) The pellicle included more proteins with an almost coiled structure, but no proteins consisting only alpha helices. Overall, salivary proteins were more organized. Categories: “helical/strand/coil” = >90% of amino acids are predicted to be organized in this motif; “majorly helical/majorly strand/majorly coil”  =  50–90% are predicted to be included in these motives; “mixed” = <50% of amino acids are belonging to one type of secondary structure motif. (**c**) The frequency of exposed or buried amino acids did also not differ significantly (pellicle: white; saliva: grey). (**d**) Both proteomes (pellicle: white; saliva: grey) had comparable distribution of molecular shapes. A score of 1 indicates a perfectly spherical shape, higher scores indicate more stretched shapes. AU  =  arbitrary units (**e**) Quaternary structure organization was more complex for salivary proteins. The pellicle included more proteins with fewer repeats and subunits. Salivary proteins are more often organized in larger complexes of up to 14 subunits and 12 repeats. Statistical comparison of proteomes was performed using Mann-Whitney-U test.

We further explored the secondary structure of pellicle and salivary proteins. In a first approach, the relative amount of alpha helices, beta strands and coiled structures was calculated, revealing no significant differences between the two proteomes (Fig. 4a; p > 0.5003). Most proteins in both proteomes showed one dominant structural motive or a prevailing coiled structure (Fig. 4b). The overall solvent accessible molecular surface was highly comparable between salivary proteins and pellicle proteins (Fig. 4c). Around 30% of the residues were exposed and 30% were buried in the inner side of the molecules.

We further obtained three-dimensional data of proteins and calculated a shape score for each molecule, where a score of one reflects an exactly round molecule, while higher scores represent more stretched shapes. There was no significant difference in shape distribution between both proteomes (p = 0.202; Fig. 4d).

Finally, we assessed the quaternary protein structures and grouped available three-dimensional structures (Fig. 4e). Pellicle proteins consisted of fewer subunits arranged in fewer repeats than salivary proteins, which is in accordance with pellicle proteins being generally smaller.

### Functionality and interactions

**Figure 5:**
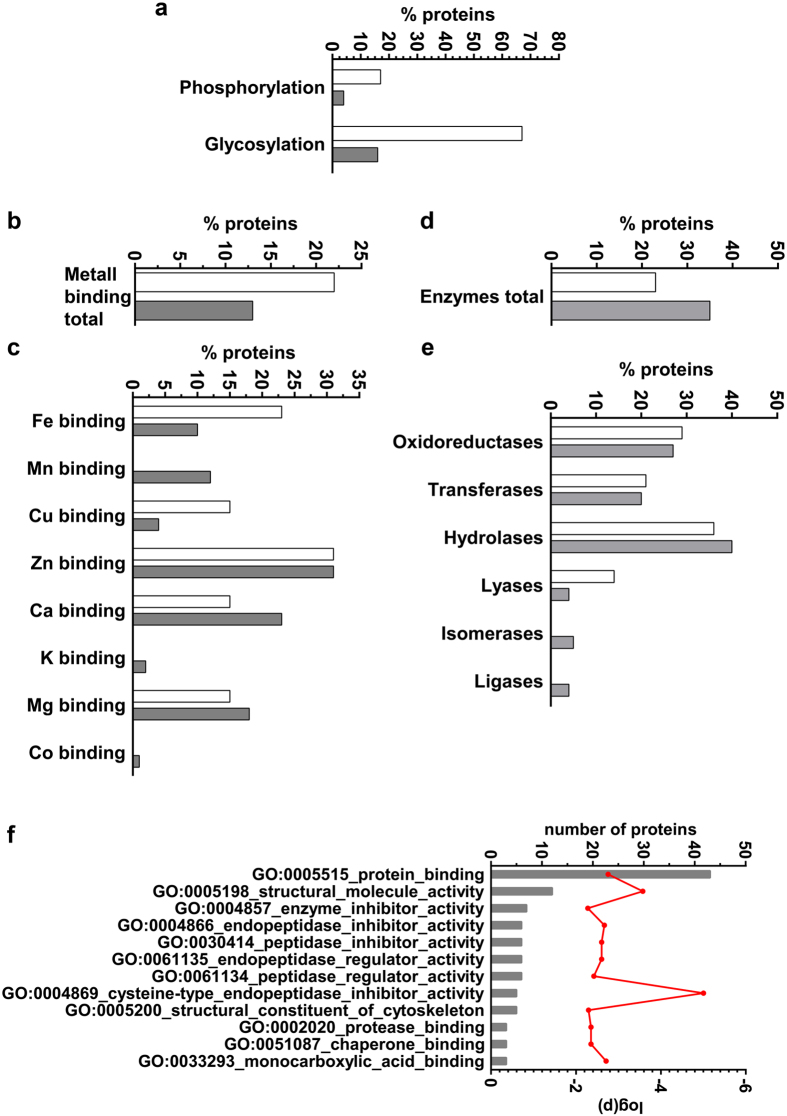
Post-translational modification, metal ion binding and enzymatic functions of pellicle and salivary proteins. **(a)** Proteins of the pellicle (white) were more often phosphorylated and glycosylated (p < 0.0001). **(b,c)** Potential to bind to metals differed between both proteomes. Binding of cobalt, potassium and manganese was limited to salivary proteins (grey). **(d,e)** Proportion of proteins with enzymatic function. The salivary proteome (grey) included all six main enzyme classes. Pellicle (white) enzyme functions were limited to oxidoreductases, transferases, hydrolases and lyases. **(f)** Gene ontology enrichment analysis reveals functional differences between the proteome of the pellicle and the saliva. The proteins in the pellicle were enriched for enzyme regulatory functions and included a higher potential to bind to other proteins (p = 0.0016). Protein counts per category is represented by grey bars, the red line indicates calculated p-values for each ontology. Mann-Whitney-U test was archived for statistical comparison.

Post-translation modification by phosphorylation and glycosylation (Fig. 5a) was significantly more common in pellicle than salivary proteins (p < 0.0001). Binding of metal ions showed different patterns in pellicle than salivary proteins, while these differences remained statistically non-significant (p = 0.4056; Fig. 5b). Detailed analysis showed higher binding capability for iron and copper in pellicle than salivary proteins, while calcium and magnesium binding was more common in salivary than pellicle proteins. Binding of manganese, potassium and cobalt was limited to salivary proteins only, whereas binding to zinc was reported for 30% of metal binding proteins in both datasets (Fig. 5c).

As most of these ions act as enzymatic co-factors, we investigated how often different enzyme classes were found in each proteome (Fig. 5d). 35% of the salivary proteins showed an enzymatic activity, while in the pellicle this proportion was 23% (p = 0.0535). Clustering for the main enzyme classes found a comparable content of oxidoreductases, transferases and hydrolases in both proteomes. In contrast, the pellicle included more lyases, while isomerases and ligases were limited to the salivary proteome (Fig. 5e). Functions of these salivary-specific enzymes included cis-trans-isomerases (EC 5.2.x.x.), intramolecular oxidoreductases (EC 5.3.x.x), intramolecular transferases (EC 5.4.x.x), enzymes forming carbon-oxygen bonds (EC 6.1.x.x), carbon-sulfur bonds (EC 6.2.x.x) or carbon-nitrogen bonds (EC 6.3.x.x).

We compared gene ontology (GO) annotations for both proteomes to decipher differences in functionality (Fig. 5f). Compared to the salivary proteome, the pellicle proteome included significantly more enriched functions for enzyme activation and inhibition combined with the potential to bind protein structures (p = 0.0016). The strongest enrichment was found for cysteine-type endopeptidase inhibitor activity, whereas more than 40 proteins were annotated as protein binders.

**Figure 6:**
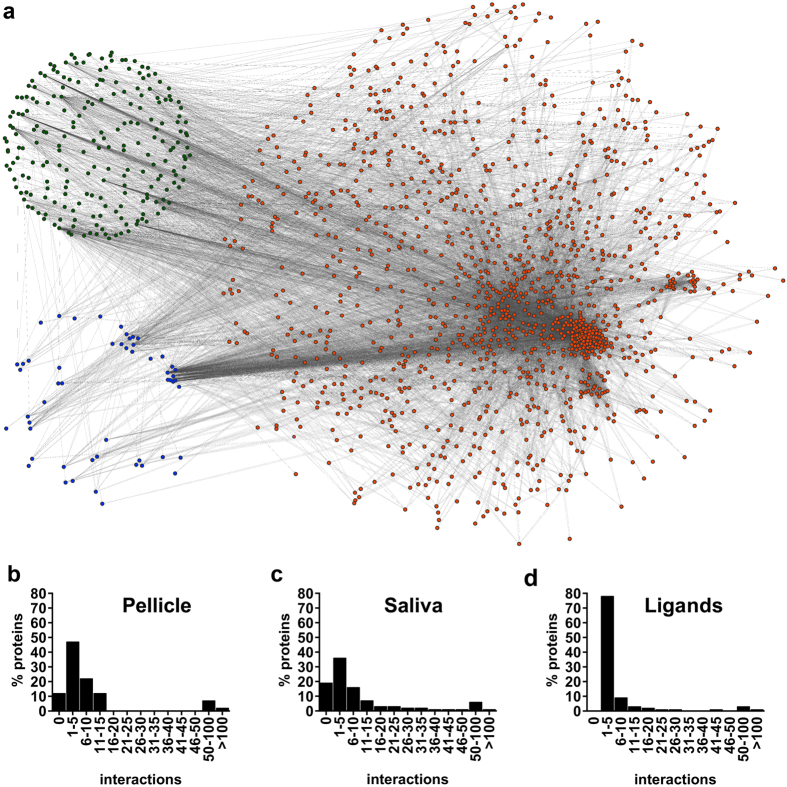
The interactome between pellicle and salivary proteins and ligands. **(a)** Overview of the interactome representing 1,220 salivary proteins (orange), 53 pellicle proteins (blue) and 223 ligands (green). Protein-protein interactions were extracted from the literature and ligand interactions predicted. **(b–d)** The majority of proteins and ligands showed 1–5 or 5–10 interactions.

Protein-protein interactions (PPIs) in both proteomes were extracted from recently published datasets^16^,^17^,^18^,^19^, resulting in 21,058 entries. Of these, 8,907 duplicates were removed and 538 self-interactions excluded for better visualization, yielding a final set of 11,613 protein-protein interactions (between a total of 1,273 proteins). The resulting PPI analysis included 88.3% of the proteins of the pellicle and 80.5% of the salivary proteins; for the remaining proteins, no interactions had been reported. In addition, 243 interactions with non-pellicle non-salivary ligands were predicted, yielding 1,918 additional interactions (Fig. 6a).

**Figure 7:**
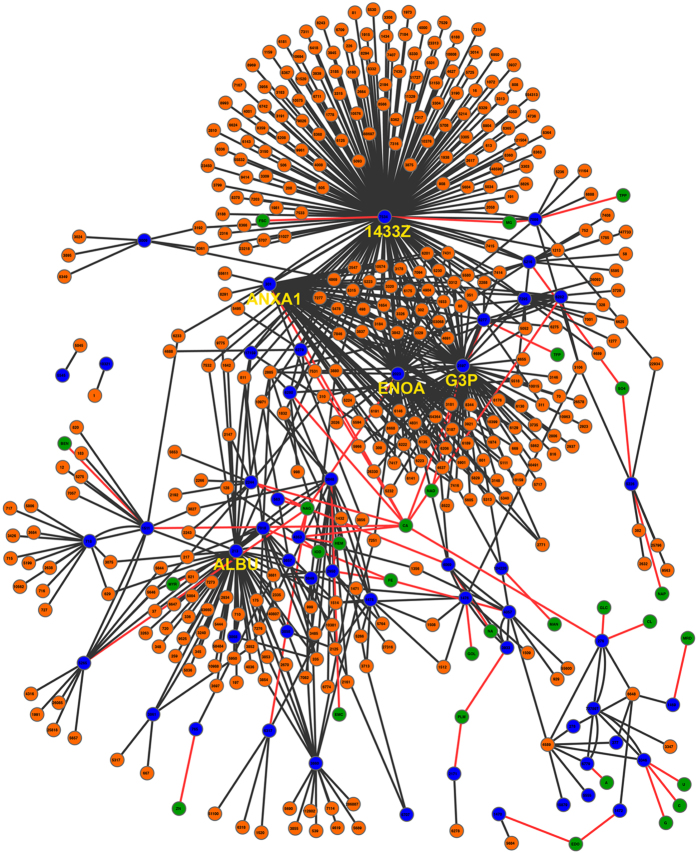
Interactions of the pellicle proteins. The 53 proteins of the pellicle (blue) interact with 405 salivary proteins (orange) and 28 ligands (green).The interactome is highly dominated by 5 proteins (highlighted by protein name), which show >50 interactions each. Interactions extracted from external sources are dark grey, predicted protein-ligand interactions are shown by red lines. Each protein is specified by its gene ID. Ligand IDs are based on Protein Data Bank ligand abbreviations (www.rcsb.org). A high resolution version of this figure is available in the online supplement. ALBU = Serum albumin, ANXA1 = Annexin A1, ENOA = Alpha-enolase, G3P = Glyceraldehyde-3-phosphate dehydrogenase, 1433Z = 14-3-3 protein zeta/delta.

Interconnectivity was similar between proteomes with a mean of 16 (1/264) (mean [min/max]) and 12 (1/184) interactions per protein in the saliva and pellicle proteome, respectively (Fig. 6b–d). When investigating the full pellicle interactome (Fig. 7), we found 732 interactions (5.4% of all identified interactions), 405 of them with salivary proteins (26.7% of all salivary proteins) and 48 with potential ligands (11.5% of all ligands). The five most connected proteins (>50 interactions) of the pellicle were Serum albumin, Annexin A1, Alpha-enolase, Glyceraldehyde-3-phosphate dehydrogenase and 14–3–3 protein zeta/delta. The most frequent observed ligands were calcium, N-acetylglucosamine and iron. In agreement with the results of the GO-enrichment analysis, the most frequently interacting proteins harbored enzymatic regulatory functions, enzymatic activity and protein binding potential.

## Discussion

The generation and comparison of data on protein sequences greatly enhances the understanding of tissue-specific protein function in health and disease^20^. Linking different datasets with each other and applying a range of bioinformatic analysis tools allows to generate a reliable and valid database, and to move from pure molecular description of single proteins to systems biology and the identification of key proteins for possible medical applications^21^,^22^. For example, data from different repositories and sources were combined to define the core proteins of the human proteome, with GO annotation being used to identify signaling sequences for protein (re)localization and molecular organization^15^,^23^,^24^. Similarly, biomarkers for pregnancy-associated abnormalities were comprehensively assessed using such datamining and comparison approach^25^, as was the human sperm proteome^26^. Given the biological but also medical relevance and potential of human saliva^27^, a comprehensive and structured analysis of salivary and pellicle proteome was needed, too.

The present study used such approach, combining a systematic review with bioinformatic analyses. We found specific differences between pellicle and the salivary proteins, but also confirmed that proteomes were rather similar in many aspects (as one could expect given that pellicle proteome constituting a subgroup of the salivary proteome).

Surprisingly, the proteomes did not differ significantly regarding their physico-chemical properties, molecular organization or solvent accessible surface; the only difference in this regard was molecular size distribution, with pellicle proteins being significantly smaller and shorter. The latter might be, as recruiting of salivary proteins to the enamel surface is a selective process^12^,^13^ which is influenced by protein weight and shape^4^. This selectivity might be increased by further post-translational modification and process-regulation (for example by phosphorylation of Serine, increasing the protein bond strength to hydroxyapatite^28^). We observed higher grade of phosphorylation (and glycosylation) in our analysis supporting the aforementioned selective regulation of molecular affinity to tooth enamel.

This finding could be relevant for potential therapeutical applications. Additionally, the pellicle proteins showed a higher net charge under extreme pH-conditions, which leads to a higher buffer capacity in very acidic (and also very alkaline) milieus. This increases the ionic interaction strength of proteins binding to hydroxyapatite surfaces and protects the enamel against acidic attacks^4^. Saliva proteins do not need to provide such effective buffering because this is maintained by soluble ions in the saliva. In contrast, saliva proteins need protection against uncontrolled denaturation and decreased affinity to oral surfaces, which both is likely realized by proteins being larger in the saliva (than the pellicle).

Moreover, we found the pellicle proteome to include significantly more enzymes than the salivary proteome, with higher enzymatic activity for lyases and isomerases and higher inhibitory function for proteases in the pellicle. Functionality of the pellicle is likely to be maintained by enzymatic activity including cross-linking and amino acid side chain modifications, while destructive mechanisms via proteases/peptidases are reduced^3^,^29^,^30^. The described side chain modifications with sugar or phosphate residues may serve as dominant substrates during pellicle maturation. The most enriched molecular function, cysteine-type endopeptidase inhibitor activity also supports this scenario: Endopeptidases are key enzymes for protein degradation in mammals^31^. In a growing proteinous layer this function is counter-productive; enzyme inhibitor activity might thus be needed to manifest a stable and functional pellicle.

Beside the molecular size and enzymatic differences, the differential potential to bind to other proteins might contribute to the functionality of the pellicle. A high cross-linking potential has been revealed for pellicle proteins, with >75% commanding the potential to bind other proteins. This specific feature aids the construction of structured and functional protein layers, but also assists to organize dental biofilm^11^. Such protein-protein-complexes can further serve as anti-erosive protection of the enamel^2^,^32^. We identified five proteins interacting with >50 salivary and/or pellicle proteins and which represent 59% of all discovered PPIs: Alpha-enolase and Glyceralaldehyde-3-phosphate dehydrogenase are well known enzymes in glycolysis and act as mandatory proteins in metabolism in numerous tissues and body fluids. Our analysis also identified Serum albumin and Annexin A1 as key hubs in the pellicle interactome, both are relevant for binding various ions (which could assist dental hard tissue remineralization). The most interacting protein was 14–3–3 zeta/delta, which has been identified as multi-adapter protein implicated in regulation of general and specialized signaling pathways by binding and modulation the activity of the binding partner. All five proteins are relevant targets for diagnostic applications^7^,^33^,^34^ but could also be relevant therapeutically, e.g. for dental biofilm management. Targeting these or other specific protein domains via administration of specifically modified, probiotic bacteria with high affinity to these structures might be feasible. Another therapeutic application might be to modify and improve the pellicle structure and function towards anti-bacterial or anti-biofilm adhesion properties. In general, the identified specific structure and functionality of the pellicle compared with the saliva might be useful for designing selective pharmaceutical drugs. while a more specific analysis of both proteomes in health and disease is likely to be useful to identify biomarkers for individualized prognosis and therapy decision^9^,^35^.

Mass spectrometry based protein identification has become the gold-standard for proteome analyses^35^, but is prone for technical and inter-individual variations, impacting on reproducibility between laboratories^36^,^37^. The present study found published datasets from single studies on the salivary and pellicle proteome to have limited agreement. Combining several datasets in a comprehensive database after systematic review and using a strict cut-off criterion significantly increased this agreement and yielded a reproducible basis for the investigation of proteomes. The deduced amino acid distribution is in corroboration with available experimental data^38^,^39^,^40^,^41^, which confirms our *in silico* analytic approach to be valid.

This study has a number of limitations. First, the applied stringency cut-off increased the reliability of the constructed database, but is prone for information loss and decreased sensitivity. This also reduced the number of included proteins, especially in the pellicle proteome, resulting in limited statistical power for many comparisons. Statistical non-significance should thus not be confused with biological non-difference. Second, this study was not deductive, i.e. hypothesis-testing, but explorative. The approach of datamining is prone for false-positive findings, which is why we established a stricter level of significance, accounting for the possible alpha-inflation. Third, the established salivary proteome certainly consists proteins originated from blood, serum, epithelia and microorganisms, most of which are not secreted by the salivary glands^33^,^34^. Including these proteins was justified given that the resulting whole saliva is the true physiological body fluid. Fourth, both biological inter-individual variance and technical aspects like sample collection method and time will impact on the resulting set of proteins identified by each study^42^. In line with this, post-translational modifications will vary between experimental conditions. *In silico* analyses are useful to investigate how different environmental conditions could theoretically impact on protein modification, structure and functionality. High-throughput data are needed to conclude more firmly on any external regulation of amino acid modifications or any cross-talk between amino acids and proteins.

Future studies should aim to combine the yielded proteomic data with that from salivary microbial metabolome studies^43^,^44^. Understanding the interaction between the human and bacterial (surface) proteins as well as soluble metabolites will help to identify critical steps in pellicle growth, biofilm maturation and pathogenic shift of the oral milieu. Additionally, relevant binding motifs or sensitive time frames for potential therapeutic interventions may be revealed.

In conclusion, the present study investigated the differences between proteins of the acquired enamel pellicle and proteins of saliva. We provided a comprehensive data resource for both proteomes based on experimental data and, to our best knowledge, performed the first functional analysis of both protein sets to identify specific molecular or functional features, which may serve as potential targets for diagnostic or therapeutic applications.

## Material and Methods

### Literature Search

The systematic literature search on proteomic data was performed in February 2015. We included observational studies which reported on at least 15 different proteins identified by mass-spectrometry in whole unstimulated saliva and/or dental pellicle originated from whole unstimulated saliva of healthy humans. Only articles published 2000 or later were considered, as the described kind of studies were unlikely to have been published earlier. Only peer-reviewed publications were considered. No language or quality restrictions were applied. The outcome parameter was reported proteins.

We searched Medline via PubMed, Embase via DIMDI, Google Scholar and opengrey.eu using the search terms specified in Supplemental Fig. 3. References of identified full-texts were screened and cross-referenced, and existing reviews on combined protein data were assessed. We planned to contact study authors if required to obtain full-texts or datasets.

One reviewer (HS) screened all titles for inclusion of full texts. A second reviewer (MW) re-screened databases for potential misses to increase sensitivity. Full-texts were then assessed independently by both reviewers after de-duplication. Studies were included in agreement, no disagreement between reviewers occured.

### Database generation and validation

Identified protein IDs were extracted independently by two reviewers (HS, MW). Only reviewed entries of the Uniprot database (Uniprot release 2014_11) were included. David toolbox (David Bioinformatics Resources 6.7)^45^ was used to convert International Protein Index (IPI) to Uniprot IDs. Non-human proteins based on Uniprot annotations were removed. Reported Uniprot IDs were manually updated to the latest release of the database by one reviewer (HS) and the resulting database was re-checked for the aforementioned ID criteria by a second reviewer (FS), who confirmed the constructed database.

To yield a robust and reliable database, a stringency cut-off for inclusion in the final database was set, including only proteins with minimum three independent experimental identifications. Validation of the final database was performed by comparing included and excluded proteins with those identified by studies of the salivary gland proteome (as the salivary glands are the major source of both salivary and pellicle proteins). Salivary gland proteins were identified using The Human Protein Atlas (Version 13 using Ensembl version 75.37)^14^ and Proteomics database resources^15^. We also checked for any indication of selection bias via molecular weight and reported signal intensity as per mass spectrometric identifications^15^.

### Comparison of proteomes

Protein/gene annotations were obtained from Uniprot (Uniprot release 2014_11). Final datasets for saliva and pellicle are available in the supplemental material of the article. To obtain the described protein properties, the following online resources were used: RaptorX Webserver^46^,^47^, EMBOSS iep Webserver^48^, Netsurfp prediction tool^49^, CAMP Database^50^, Periodic Table of Protein Complexes^51^ and GO miner^52^. We needed to use a range of bioinformatics tools as no single tool allows to comprehensively analyze all relevant parameters. A summary of the obtained results including dates of request, detailed settings and link to the web server are available in the supplementary information (Supplementary Tab. 2). Available three-dimensional data of the proteins were obtained from the Protein Data Bank (PDB)^53^ and used to calculate the described shape score.

Protein-protein-interactions were obtained from recently published mass spectrometry studies^16^,^17^,^18^,^19^. Protein-ligand interactions were predicted based on the RaptorX ligand binding prediction^46^. Interactions with prediction scores >30 were considered, which is in line with recommendations of the developers (note that we decreased the recommended threshold from 40 to 30 to increase the sensitivity of our analysis)^46^,^47^.

For validation, a subset of those ligands (including adenosine monophosphate, adenosine triphosphate, calcium, flavin adenine dinucleotide, iron, flavin mononucleotiode, guanosine triphosphate, potassium, magnesium, manganese, nicotinaminde-adenine-dinucleotide, nicotinamide-adenine-dinucleotide phosphate, p-nitrophenol, thiamine diphosphate and zinc; representing 26.97% [n = 518] of all predicted ligand interactions) was compared with available binding data from Uniprot. For 22.78% of the predicted interactions we found experimental evidence in the Uniprot data repository. All interactions were plotted using Cytoscape 2.8.2.

Statistical comparison of proteomes was performed using Mann-Whitney-U test or Fisher’s exact test. Level of significance was adjusted for multiple testing using the Bonferroni correction.

## Additional Information

**How to cite this article**: Schweigel, H. *et al*. Salivary and pellicle proteome: A datamining analysis. *Sci. Rep.* **6**, 38882; doi: 10.1038/srep38882 (2016).

**Publisher's note:** Springer Nature remains neutral with regard to jurisdictional claims in published maps and institutional affiliations.

## Supplementary Material

Supplementary Information
