# Supplementary material for: Salivary and pellicle proteome: A datamining analysis
Source: Sci Rep. 2016 Dec 14;6:38882. doi: 10.1038/srep38882 (PMC5155218; doi:10.1038/srep38882)
Supplement: Supplementary Information [file srep38882-s1.doc]

***Salivary and pellicle proteome: A datamining analysis***

Hardy Schweigel^1^, Michael Wicht^2^ and Falk Schwendicke^3 #^

^1^ Department of Clinical Research, DMG Dental-Material Gesellschaft, Hamburg, Germany

^2^ Polyclinic of Operative Dentistry and Periodontology, University of Cologne, Kerpener Straße 32, 50931 Cologne

^3^ Operative and Preventive Dentistry, Charité – Universitätsmedizin Berlin, Berlin, Germany

**Supplement files**


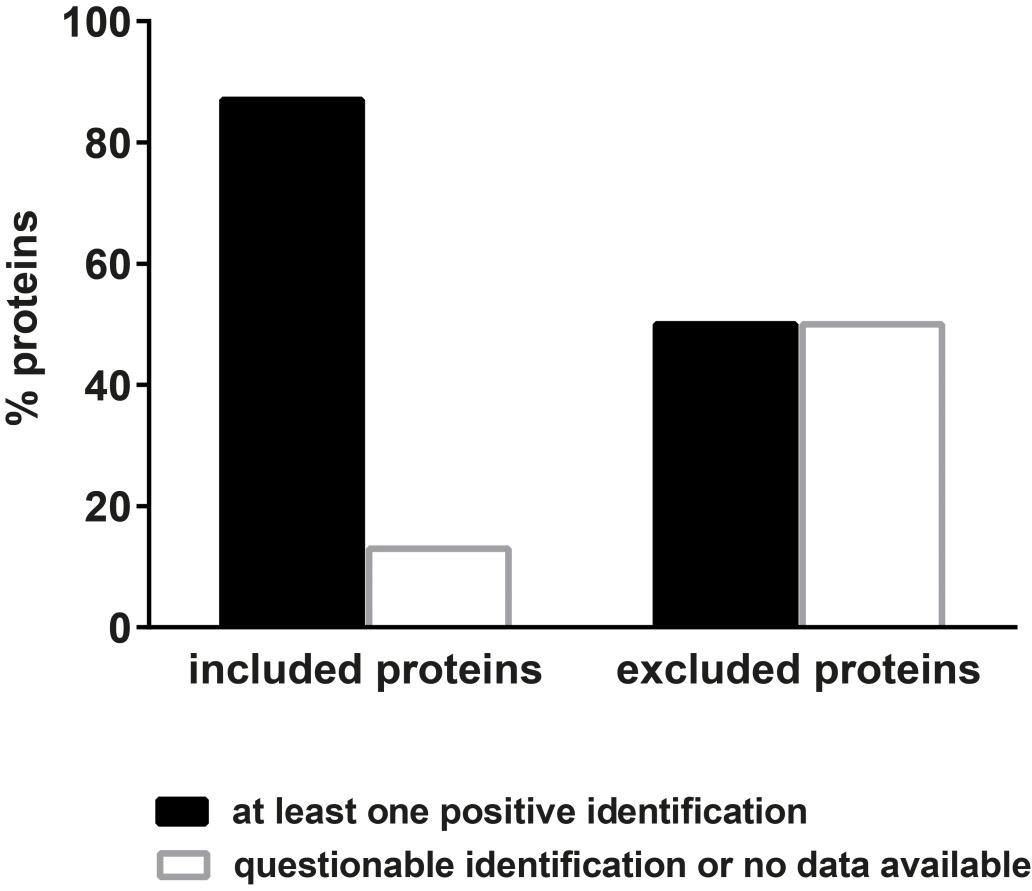


**Supplemental figure 1:** **Validation of included proteins by comparison with the reported proteome of the salivary glands, identified via immunohistology or mass spectrometry.** Almost 90% of included proteins were also identified in salivary glands using one or both methods. This was not the case for excluded proteins.


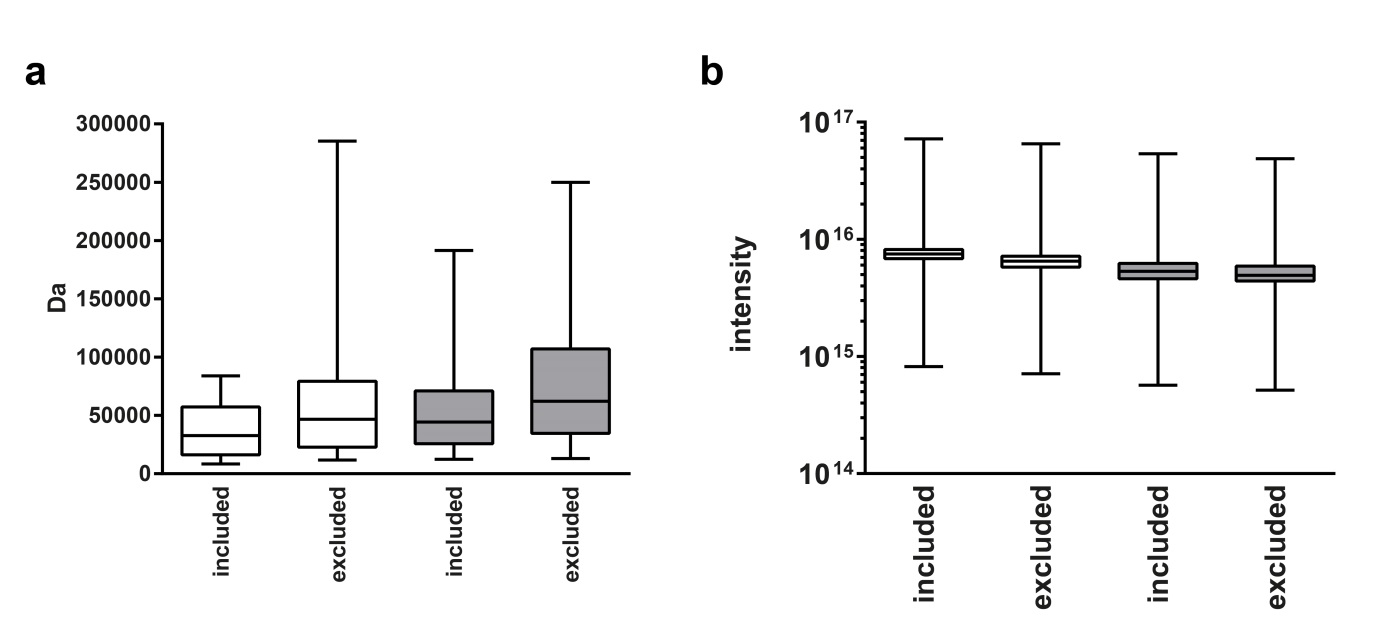


**Supplemental figure 2:** **Analysis for potential selection bias of included proteins.** **(a-b)** There was no difference in the distribution of molecular weight and mass spectrometric signal intensity between included and excluded proteins (pellicle: white boxes; saliva: grey boxes).


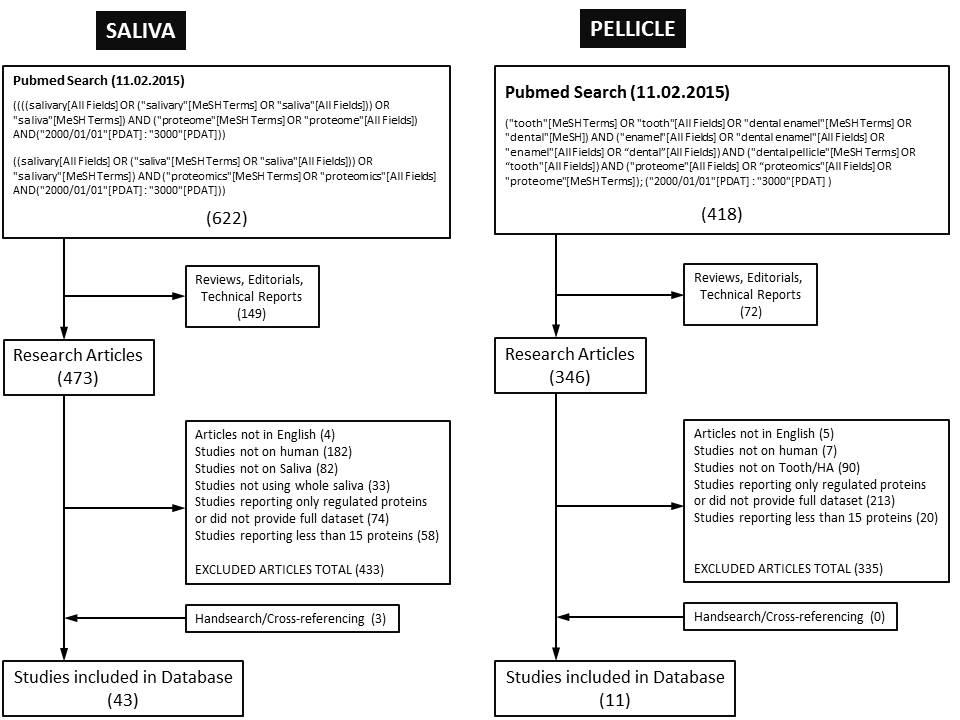


**Supplemental figure 3: Summary of literature search strategy and article extraction.** The search strategy is shown for Medline via PubMed and was adapted for other databases.

**Table S1: Summary of extracted, processed and included proteins per data source.** Number of identified proteins differs from tenth to thousands after conversion of ID´s (if necessary). Manual revision for human proteins with reviewed status in the Uniprot data repository results in the preliminary global dataset with exclusion of thousands of proteins. Finally, only 1,575 proteins with at least three independent experimental identifications were considered in the final dataset reflecting 30.1% of the originally extracted proteins.

**Table S2: Overview of obtained results using several web server applications for the investigation of proteins.** For all analyses, time of request, direct link to the web server and percentage of coverage for both proteomes is summarized. Completeness of results varies and is limited by restrictions of the application itself e.g. in limiting the length of input sequences or the missing of adequate templates in the algorithm´s database for computing predictions, ligand interactions or molecular organization.


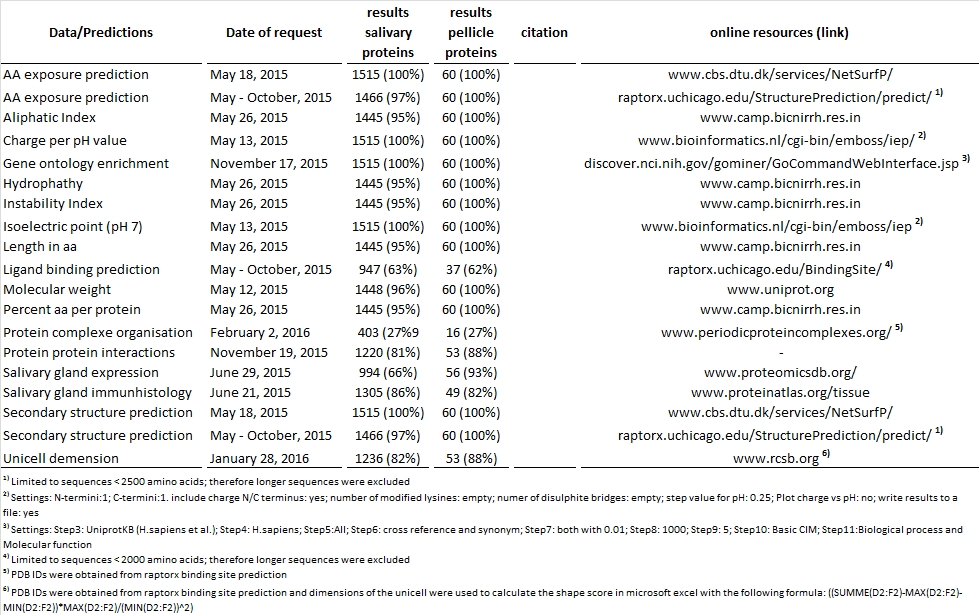


**Supplemental References**

54 Yao, Y., Berg, E. A., Costello, C. E., Troxler, R. F. & Oppenheim, F. G. Identification of protein components in human acquired enamel pellicle and whole saliva using novel proteomics approaches. *The Journal of biological chemistry* **278**, 5300-5308, doi:10.1074/jbc.M206333200 (2003).

55 Vitorino, R. *et al.* Peptidomic analysis of human acquired enamel pellicle. *Biomedical chromatography : BMC* **21**, 1107-1117, doi:10.1002/bmc.830 (2007).

56 Siqueira, W. L., Helmerhorst, E. J., Zhang, W., Salih, E. & Oppenheim, F. G. Acquired enamel pellicle and its potential role in oral diagnostics. *Annals of the New York Academy of Sciences* **1098**, 504-509, doi:10.1196/annals.1384.023 (2007).

57 Vitorino, R., Calheiros-Lobo, M. J., Duarte, J. A., Domingues, P. M. & Amado, F. M. Peptide profile of human acquired enamel pellicle using MALDI tandem MS. *Journal of separation science* **31**, 523-537, doi:10.1002/jssc.200700486 (2008).

58 Siqueira, W. L. & Oppenheim, F. G. Small molecular weight proteins/peptides present in the in vivo formed human acquired enamel pellicle. *Archives of oral biology* **54**, 437-444, doi:10.1016/j.archoralbio.2009.01.011 (2009).

59 Siqueira, W. L., Bakkal, M., Xiao, Y., Sutton, J. N. & Mendes, F. M. Quantitative proteomic analysis of the effect of fluoride on the acquired enamel pellicle. *PLoS One* **7**, e42204, doi:10.1371/journal.pone.0042204 (2012).

60 Zimmerman, J. N. *et al.* Proteome and peptidome of human acquired enamel pellicle on deciduous teeth. *International journal of molecular sciences* **14**, 920-934, doi:10.3390/ijms14010920 (2013).

61 Lee, Y. H. *et al.* Proteomic evaluation of acquired enamel pellicle during in vivo formation. *PLoS One* **8**, e67919, doi:10.1371/journal.pone.0067919 (2013).

62 Masson, N., Domingues, R. R., Cury, J. A. & Paes Leme, A. F. Acidulated phosphate fluoride application changes the protein composition of human acquired enamel pellicle. *Caries research* **47**, 251-258, doi:10.1159/000346280 (2013).

63 Ash, A., Mulholland, F., Burnett, G. R. & Wilde, P. J. Structural and compositional changes in the salivary pellicle induced upon exposure to SDS and STP. *Biofouling* **30**, 1183-1197, doi:10.1080/08927014.2014.977268 (2014).

64 Delecrode, T. R. *et al.* Identification of acid-resistant proteins in acquired enamel pellicle. *Journal of dentistry* **43**, 1470-1475, doi:10.1016/j.jdent.2015.10.009 (2015).

65 Ghafouri, B., Tagesson, C. & Lindahl, M. Mapping of proteins in human saliva using two-dimensional gel electrophoresis and peptide mass fingerprinting. *Proteomics* **3**, 1003-1015, doi:10.1002/pmic.200300426 (2003).

66 Vitorino, R. *et al.* Identification of human whole saliva protein components using proteomics. *Proteomics* **4**, 1109-1115, doi:10.1002/pmic.200300638 (2004).

67 Wilmarth, P. A. *et al.* Two-dimensional liquid chromatography study of the human whole saliva proteome. *Journal of proteome research* **3**, 1017-1023, doi:10.1021/pr049911o (2004).

68 Xie, H., Rhodus, N. L., Griffin, R. J., Carlis, J. V. & Griffin, T. J. A catalogue of human saliva proteins identified by free flow electrophoresis-based peptide separation and tandem mass spectrometry. *Molecular & cellular proteomics : MCP* **4**, 1826-1830, doi:10.1074/mcp.D500008-MCP200 (2005).

69 Hirtz, C. *et al.* MS characterization of multiple forms of alpha-amylase in human saliva. *Proteomics* **5**, 4597-4607, doi:10.1002/pmic.200401316 (2005).

70 Hu, S. *et al.* Human saliva proteome and transcriptome. *Journal of dental research* **85**, 1129-1133 (2006).

71 Walz, A. *et al.* Proteome analysis of glandular parotid and submandibular-sublingual saliva in comparison to whole human saliva by two-dimensional gel electrophoresis. *Proteomics* **6**, 1631-1639, doi:10.1002/pmic.200500125 (2006).

72 Guo, T. *et al.* Characterization of the human salivary proteome by capillary isoelectric focusing/nanoreversed-phase liquid chromatography coupled with ESI-tandem MS. *Journal of proteome research* **5**, 1469-1478, doi:10.1021/pr060065m (2006).

73 Harthoorn, L. F. *et al.* Salivary biomarkers associated with perceived satiety and body mass in humans. *Proteomics. Clinical applications* **1**, 1637-1650, doi:10.1002/prca.200700448 (2007).

74 Fang, X. *et al.* Comparison of electrokinetics-based multidimensional separations coupled with electrospray ionization-tandem mass spectrometry for characterization of human salivary proteins. *Analytical chemistry* **79**, 5785-5792, doi:10.1021/ac070611a (2007).

75 Ohshiro, K. *et al.* Pre-analytic saliva processing affect proteomic results and biomarker screening of head and neck squamous carcinoma. *Int J Oncol* **30**, 743-749 (2007).

76 Esser, D. *et al.* Sample Stability and Protein Composition of Saliva: Implications for Its Use as a Diagnostic Fluid. *Biomark Insights* **3**, 25-27 (2008).

77 Denny, P. *et al.* The proteomes of human parotid and submandibular/sublingual gland salivas collected as the ductal secretions. *Journal of proteome research* **7**, 1994-2006, doi:10.1021/pr700764j (2008).

78 Hu, S., Loo, J. A. & Wong, D. T. Human saliva proteome analysis. *Annals of the New York Academy of Sciences* **1098**, 323-329, doi:10.1196/annals.1384.015 (2007).

79 Rao, P. V. *et al.* Proteomic identification of salivary biomarkers of type-2 diabetes. *Journal of proteome research* **8**, 239-245, doi:10.1021/pr8003776 (2009).

80 Fleissig, Y. *et al.* Different proteomic protein patterns in saliva of Sjogren's syndrome patients. *Oral Dis* **15**, 61-68, doi:10.1111/j.1601-0825.2008.01465.x (2009).

81 Sun, X., Salih, E., Oppenheim, F. G. & Helmerhorst, E. J. Activity-based mass spectrometric characterization of proteases and inhibitors in human saliva. *Proteomics. Clinical applications* **3**, 810-820, doi:10.1002/prca.200800242 (2009).

82 Vitorino, R. *et al.* Towards defining the whole salivary peptidome. *PROTEOMICS – Clinical Applications* **3**, 528-540, doi:10.1002/prca.200800183 (2009).

83 Jessie, K., Pang, W. W., Haji, Z., Rahim, A. & Hashim, O. H. Proteomic analysis of whole human saliva detects enhanced expression of interleukin-1 receptor antagonist, thioredoxin and lipocalin-1 in cigarette smokers compared to non-smokers. *International journal of molecular sciences* **11**, 4488-4505, doi:10.3390/ijms11114488 (2010).

84 Bandhakavi, S., Stone, M. D., Onsongo, G., Van Riper, S. K. & Griffin, T. J. A dynamic range compression and three-dimensional peptide fractionation analysis platform expands proteome coverage and the diagnostic potential of whole saliva. *Journal of proteome research* **8**, 5590-5600, doi:10.1021/pr900675w (2009).

85 de Jong, E. P. *et al.* Quantitative proteomics reveals myosin and actin as promising saliva biomarkers for distinguishing pre-malignant and malignant oral lesions. *PLoS One* **5**, e11148, doi:10.1371/journal.pone.0011148 (2010).

86 Fleissig, Y. *et al.* Comparative proteomic analysis of human oral fluids according to gender and age. *Oral Dis* **16**, 831-838, doi:10.1111/j.1601-0825.2010.01696.x (2010).

87 Krief, G. *et al.* Improved visualization of low abundance oral fluid proteins after triple depletion of alpha amylase, albumin and IgG. *Oral Dis* **17**, 45-52, doi:10.1111/j.1601-0825.2010.01700.x (2011).

88 Krief, G. *et al.* Comparison of diverse affinity based high-abundance protein depletion strategies for improved bio-marker discovery in oral fluids. *J Proteomics* **75**, 4165-4175, doi:10.1016/j.jprot.2012.05.012 (2012).

89 Jagtap, P. *et al.* Workflow for analysis of high mass accuracy salivary data set using MaxQuant and ProteinPilot search algorithm. *Proteomics* **12**, 1726-1730, doi:10.1002/pmic.201100097 (2012).

90 Vitorino, R., Guedes, S., Manadas, B., Ferreira, R. & Amado, F. Toward a standardized saliva proteome analysis methodology. *J Proteomics* **75**, 5140-5165, doi:10.1016/j.jprot.2012.05.045 (2012).

91 Zauber, H., Mosler, S., Heßberg, A. v. & Schulze, W. X. Dynamics of salivary proteins and metabolites during extreme endurance sports – a case study. *Proteomics* **12**, 2221-2235, doi:10.1002/pmic.201100228 (2012).

92 Jehmlich, N. *et al.* Quantitative analysis of the intra- and inter-subject variability of the whole salivary proteome. *Journal of periodontal research* **48**, 392-403, doi:10.1111/jre.12025 (2013).

93 Salazar, M. G. *et al.* Identification of periodontitis associated changes in the proteome of whole human saliva by mass spectrometric analysis. *Journal of clinical periodontology* **40**, 825-832, doi:10.1111/jcpe.12130 (2013).

94 Dominy, S. S. *et al.* Proteomic analysis of saliva in HIV-positive heroin addicts reveals proteins correlated with cognition. *PLoS One* **9**, e89366, doi:10.1371/journal.pone.0089366 (2014).

95 Goncalves Lda, R., Campanhon, I. B., Domingues, R. R., Paes Leme, A. F. & Soares da Silva, M. R. Comparative salivary proteome of hepatitis B- and C-infected patients. *PLoS One* **9**, e113683, doi:10.1371/journal.pone.0113683 (2014).

96 Ngounou Wetie, A. G. *et al.* A Pilot Proteomic Analysis of Salivary Biomarkers in Autism Spectrum Disorder. *Autism Res* **8**, 338-350, doi:10.1002/aur.1450 (2015).

97 de Sousa-Pereira, P. *et al.* Cross-species comparison of mammalian saliva using an LC–MALDI based proteomic approach. *Proteomics* **15**, 1598-1607, doi:10.1002/pmic.201400083 (2015).

98 Salih, E., Siqueira, W. L., Helmerhorst, E. J. & Oppenheim, F. G. Large-scale phosphoproteome of human whole saliva using disulfide-thiol interchange covalent chromatography and mass spectrometry. *Analytical biochemistry* **407**, 19-33, doi:10.1016/j.ab.2010.07.012 (2010).

99 Stone, M. D. *et al.* Large-scale phosphoproteomics analysis of whole saliva reveals a distinct phosphorylation pattern. *Journal of proteome research* **10**, 1728-1736, doi:10.1021/pr1010247 (2011).

100 Ramachandran, P. *et al.* Identification of N-linked glycoproteins in human saliva by glycoprotein capture and mass spectrometry. *Journal of proteome research* **5**, 1493-1503, doi:10.1021/pr050492k (2006).

101 Larsen, M. R., Jensen, S. S., Jakobsen, L. A. & Heegaard, N. H. Exploring the sialiome using titanium dioxide chromatography and mass spectrometry. *Molecular & cellular proteomics : MCP* **6**, 1778-1787, doi:10.1074/mcp.M700086-MCP200 (2007).

102 Ramachandran, P. *et al.* Comparison of N-linked Glycoproteins in Human Whole Saliva, Parotid, Submandibular, and Sublingual Glandular Secretions Identified using Hydrazide Chemistry and Mass Spectrometry. *Clinical proteomics* **4**, 80-104, doi:10.1007/s12014-008-9005-0 (2008).

103 Ferreira, J. A. *et al.* Synthesis and optimization of lectin functionalized nanoprobes for the selective recovery of glycoproteins from human body fluids. *Analytical chemistry* **83**, 7035-7043, doi:10.1021/ac200916j (2011).

104 Sondej, M. *et al.* Glycoprofiling of the Human Salivary Proteome. *Clinical proteomics* **5**, 52-68, doi:10.1007/s12014-008-9021-0 (2009).

105 Bandhakavi, S. *et al.* Hexapeptide libraries for enhanced protein PTM identification and relative abundance profiling in whole human saliva. *Journal of proteome research* **10**, 1052-1061, doi:10.1021/pr100857t (2011).

106 Sun, S. *et al.* Analysis of age and gender associated N-glycoproteome in human whole saliva. *Clinical proteomics* **11**, 1-10, doi:10.1186/1559-0275-11-25 (2014).
